# Bayesian cognitive science, predictive brains, and the nativism debate

**DOI:** 10.1007/s11229-017-1427-7

**Published:** 2017-05-22

**Authors:** Matteo Colombo

**Affiliations:** Tilburg Center for Logic, Ethics and Philosophy of Science, Tilburg University, P.O. Box 90153, 5000 LE Tilburg, The Netherlands

**Keywords:** Bayesian cognitive science, Innateness, Connectionism, Classicism, Nativism and empiricism

## Abstract

The rise of Bayesianism in cognitive science promises to shape the debate between nativists and empiricists into more productive forms—or so have claimed several philosophers and cognitive scientists. The present paper explicates this claim, distinguishing different ways of understanding it. After clarifying what is at stake in the controversy between nativists and empiricists, and what is involved in current Bayesian cognitive science, the paper argues that Bayesianism offers not a vindication of either nativism or empiricism, but one way to talk precisely and transparently about the kinds of mechanisms and representations underlying the acquisition of psychological traits without a commitment to an innate language of thought.

## Introduction

Several philosophers and cognitive scientists believe that Bayesianism in cognitive science has novel, important consequences for the controversy between nativists and empiricists. For instance, Tenenbaum et al. (2011) claim that “the Bayesian approach lets us move beyond classic either-or dichotomies that have long shaped and limited debates in cognitive science.” One such dichotomies is *eitherempiricism or nativism* (p. 1285). Clark (2013a, b, 2016) agrees that Bayesianism “should fundamentally reconfigure our thinking about the debate between nativism and empiricism” (2013a, p. 482). Also Samet and Zaitchik (2014) seem to agree, and they single out Bayesianism as an especially relevant approach to the contemporary controversy surrounding innateness in cognitive science.

While there is agreement that Bayesianism bears on the debate between nativists and empiricists, it remains unclear just how. Existing literature in Bayesian cognitive science does not elucidate what’s exactly at stake in that debate, and philosophers who have suggested that the brain might approximately implement Bayesian inference (Hohwy 2013; Clark 2016) have not rigorously explained relevant similarities and differences between distinct approximations for Bayesian inference.

Two distinct types of ideas are conflated in the literature. The first idea is that Bayesianism matters to the debate because it bears out aspects of both nativist and empiricist views.^1^ Specifically, Bayesianism would vindicate empiricists’ emphasis on learning from experience as the central process in the acquisition of new psychological traits; but Bayesianism would also vindicate nativists’ emphasis on the role of prior knowledge in acquiring new psychological traits.

Tenenbaum et al. (2011), Clark (2013a, b, 2016), and Samet and Zaitchik (2014) can be read as having this idea in mind when they claim that Bayesianism matters to the controversy between nativists and empiricists. On the one hand, they all point out that Bayesianism offers previously unappreciated resources to empiricists, who can rely on hierarchical Bayesian modelling to show that the information required for the acquisition of high-level psychological traits need not be hardwired in a system, but can be picked up in the environmental input. On the other hand, as explained by Samet and Zaitchik (2014), Bayesianism vindicates aspects of nativism too, “because it focuses attention on the role of background knowledge in learning.” In these respects, however, Bayesianism recapitulates previous approaches like Connectionism; and so, it does not provide us with a fundamentally novel way to think about the debate between nativists and empiricists.

A distinct idea is that Bayesianism matters because it makes the debate more empirically tractable than previous approaches. Bayesianism would provide us with a distinctively precise and semantically transparent way to talk about and test how psychological traits might be acquired. Samet and Zaitchik (2014) recognize this point when they highlight that Bayesianism “provides a systematic and quantifiable approach to development.” Perfors, Tenenbaum et al. (2011, p. 317) also emphasise this virtue of Bayesianism: “its representational flexibility makes it applicable to a wide variety of learning problems, and its transparency makes it easy to be clear about what assumptions are being made, what is being learned, and why learning works.” However, while Bayesianism is more semantically transparent than an approach like Connectionism, it recapitulates Classicism in this respect; and so, it does not provide us with a fundamentally novel way to think about the debate between nativists and empiricists.

Presenting aspects of both Classicism and Connectionism, what Bayesianism brings to the table—I suggest—is a transparent way of evaluating the character of the innate structure in the human cognitive architecture without the need for a commitment to an innate language of thought. In arguing for this claim, my goal is not to defend a particular nativist or empiricist position. My goal is instead to clarify how Bayesianism can frame the debate in a more productive form.

In order to achieve my goal, I start, in Sect. 2, to clarify how the dialectical situation between nativists and empiricists should be understood. In Sects. 3 and 4, I examine the nature and role of Bayesian priors in visual perception, and show that the fact that Bayesianism posits priors does not have implications for the controversy. In Sects. 5 and 6, I focus on Bayesian learning mechanisms in categorization tasks, and show that the fact that Bayesianism posits a general-purpose learning mechanism like Bayesian conditionalization does not have implications for the controversy. In Sect. 7, I compare Bayesianism with Connectionism and Classicism, which are two prominent alternative approaches to cognitive change, and argue that Bayesianism recapitulates aspects of both Connectionism and Classicism. A short conclusion follows.

## Nativism versus Empiricism: What’s at stake?

‘Nativism’ and ‘Empiricism’ pick out broad families of views concerning the origin of psychological traits and the shape of the underlying cognitive architecture, where ‘psychological traits’ may refer to abilities, capacities, ideas, or concepts. Contemporary nativists and empiricists agree that both nature and nurture matter to questions about the origin of psychological traits. They agree that there are genetic and environmental contributions to the acquisition of psychological traits. Both sides also agree that the acquisition of psychological traits depends on a certain amount of innate structure. The disagreement concerns the character of this innate structure. It concerns the question: What kinds of mechanisms and representations in humans’ innate cognitive architecture are causally responsible for the acquisition of psychological traits? (cf., Cowie 1999, p. 26; Margolis and Laurence 2013, p. 695).

Empiricists posit as little innate endowment as possible. According to them, the innate architecture of the mind includes few general-purpose (or domain-general) mechanisms for acquiring psychological traits. Because these mechanisms are general-purpose, they operate in a wide range of different psychological domains. For example, general-purpose learning mechanisms like statistical learning and pattern recognition would operate in a wide variety of different psychological domains, and would suffice to acquiring such psychological traits as a language, knowledge of causal relations in the world, the ability to ascribe mental states to other agents, and so on (cf., Prinz 2012; on language learning and empiricism see Elman 1991; Chater et al. 2015). As these learning mechanisms are responsible for the acquisition of psychological traits by extracting statistical regularities in the environment, “whatever differentiation into domain-specific cognitive systems there might be will reflect differentiation in the [statistical structure in the] environment and not our innate endowments” (Samuels 1998, p. 576).

Nativists posit a richer innate endowment. According to them, the innate architecture of the mind includes many domain-specific mechanisms and/or bodies of knowledge for acquiring new psychological traits (Simpson et al. 2005; for a comprehensive discussion of different nativisms see Carruthers et al. 2005, 2006, 2007). Domain-specific mechanisms operate in a restricted class of problems in a narrow range of psychological domains. For example, according to some evolutionary approaches to psychology, psychological traits such as the abilities to recognize faces, ascribe mental states to others, and identify cheaters in social exchanges could be acquired only through Darwinian modules, each one of which is dedicated to operate in one psychological domain (Carruthers 2006; Pinker 2002; Sperber 1994). A domain-specific body of knowledge is a system of mental representations about a specific subject matter such as physics and psychology, which apply to a distinct domain of entities and phenomena (Carey and Spelke 1994). For example, according to some generativist approaches to linguistics, language can be acquired only because our cognitive architecture contains an innate Universal Grammar, which consists of a body of domain-specific knowledge about the grammatical principles of human natural languages and applies to sentences and their constituents (Chomsky 1980, 1988).

In summary, the disagreement between contemporary nativists and empiricists is about the kinds of learning mechanisms and representations in humans’ innate cognitive architecture. Empiricists are committed to a kind of cognitive architecture that includes few general-purpose mechanisms that are ultimately responsible for the acquisition of all psychological traits. Nativists are committed to a kind of cognitive architecture that is rich in domain-specific mechanisms and/or representations that are ultimately responsible for the acquisition of all psychological traits.

Three points of elaboration are in order. First, nativism and empiricism admit of degrees. One may be nativist (or empiricist) about a greater or smaller portion of the psychological traits in the human cognitive architecture. Second, while domain-specific bodies of knowledge may be processed by domain-specific mechanisms, this need not be so. As Samuels (1998, p. 583) explains, humans may possess a single general-purpose mechanism like a single universal Turing machine that deploys internally represented, domain-specific bodies of knowledge. Thus, one may posit innate bodies of knowledge about the principles of language, while allowing that the mechanism that recruits such representations be general-purpose (cf., Fodor 2001, pp. 106–109). Third and finally, the controversy between nativists and empiricists presupposes the legitimacy of some notion of *innateness* in cognitive science. Although *innateness* is multiply ambiguous (Mameli and Bateson 2006), may not correspond to any natural kind (Mameli and Bateson 2011), and may obscure the complexities of ontogenesis (Scholz 2002), this notion often features in discussions of the relation between Bayesian cognitive science and the nativism debate without a clear explication (cf., Samet and Zaitchik 2014, Sect. 3.2). As I shall point out below in Sect. 4, sometimes these discussions seem to assume an explication of innateness in terms of *psychological primitiveness*, where psychologically primitive traits are the ones whose acquisition cannot be explained by any adequate theory in cognitive science (Cowie 1999; Samuels 2002). Sometimes they assume an explication in terms of *developmental canalization*, which roughly corresponds to the degree of developmental rigidity of a trait in the face of variation across a range of environments (Ariew 1999; see also Mallon and Weinberg 2006). Some other time, they assume an explication of innateness in terms of *adaptation*, according to which a psychological trait is innate if its acquisition can only be explained by natural selection (cf., Lorenz 1965). Evaluating how Bayesianism matters for the debate between nativists and empiricists requires clarity about the explication of innateness one presupposes.

## Bayesian priors between nativism and empiricism

A characterisation of Bayesianism in cognitive science goes as follows. Take some problem that cognitive agents face—for example, disambiguating convex from concave shapes from shading information, or grouping objects into categories. Formulate the problem in probabilistic terms by defining a model of the process that generates some data, *d*—for example, two-dimensional retinal images, or exemplars of some underlying category. Let *H* be a set of (exhaustive and mutually exclusive) hypotheses about the process (known as *hypothesis space*). For each hypothesis $h \in \, H$, *P*(*h*) is the probability that the agent assigns to *h* being the true generating process, prior to observing the data *d*. *P*(*h*) is known as the *prior probability*. The Bayesian rule of conditionalization prescribes that, after observing data *d*, the agent should update *P*(*h*) by replacing it with $P(h {\vert } d)$ (known as the *posterior probability*). To execute the rule of conditionalization, the agent multiplies the *priorP*(*h*) by the *likelihood*$P(d {\vert } h)$ as stated by Bayes’ *theorem*:^2^

1 $$\begin{aligned} P\left( {h\hbox {|}d} \right) = \frac{P\left( {d\hbox {|}h} \right) P\left( h \right) }{\mathop \sum \nolimits _{h\in H} P\left( {d\hbox {|}h} \right) P\left( h \right) } \end{aligned}$$

where $P(d {\vert } h)$ is the probability of observing *d* if *h* were true (known as *likelihood*), and the sum in the denominator ensures that the resulting probabilities sum to one. According to (1), the posterior probability of *h* is directly proportional to the product of its prior probability and likelihood, relative to the sum of the products and likelihoods for all alternative hypotheses in the hypothesis space *H*. The rule of conditionalization prescribes that the agent should adopt the posterior $P(h {\vert } d)$ as a revised probability assignment for *h*: the new probability of *h* should be proportional to its prior probability multiplied by its likelihood.

Bayesian conditionalization alone does not specify how an agent’s beliefs should be used to generate a decision or an action. How to use the posterior distribution to generate a decision is described by Bayesian decision theory, and requires the definition of a *loss* (or utility) *functionL*(*A*, *H*). For each action $a \in \, A$—where *A* is the space of possible actions or decisions available to the agent—the loss function specifies the relative cost of taking action *a* for each possible $h \in H$. To choose the best action, the agent calculates the expected loss for each *a*, which is the loss averaged across the possible *h*, weighted by the degree of belief in *h*. The action with the minimum expected loss is the best action that the agent can take given her beliefs.

Given this characterisation of the Bayesian approach,^3^ one common suggestion is that “the key issue in considering the bearing of Bayesianism on the Nativist–Empiricist controversy is the priors” (Samet and Zaitchik 2014). The basic idea is that “[i]nnate assumptions and principles [...] are realized as priors” with certain default values that get updated via interaction with the environment (Scholl 2005, pp. 48–49).

However, because empiricists and nativists do agree that the human cognitive architecture is comprised of some innate structure, and they can also agree that this innate structure might be realized as Bayesian priors, this basic idea leaves many key issues in the debate surrounding nativism open, such as: Are all Bayesian priors evolved psychological traits, or are they psychological traits culturally acquired in cognitive development? Are all Bayesian priors psychologically primitive? Are they robust to environmental variation? Are all (or most) Bayesian priors domain-specific representations? While different answers to these more specific questions underwrite different positions in the nativism–empiricism spectrum, Bayesianism is *not* committed to positing a cognitive architecture that is rich in domain-specific representations realized as priors, which are ultimately responsible for the acquisition of all other psychological traits. To establish this claim, I now concentrate on the light-from-above prior, which is often cited as a characteristically nativist psychological trait.

## Light-from above through Bayesian lenses

The light-from-above prior is the prior “belief” that light shines from overhead (precisely, from above-left). It is often cited as an example of a characteristically nativist trait because it would be a paradigmatic example of an internally represented body of innate knowledge specific to lighting source (Hershberger 1970; Ramachandran 1988; Kersten et al. 2004, p. 285; Mamassian and Goutcher 2001; Scholl 2005; Samet and Zaitchik 2014; for a general account of priors in visual perception see Sotiropoulos and Seriès 2015).

In describing the light-from-above prior as a paradigmatic example of an innate, domain-specific representation, the literature confuses two questions.^4^ First question: Is the light-from-above prior malleable, or is it rigid? Second question: Is the light-from-above prior a psychologically primitive domain-specific representation, or can this trait be acquired courtesy of some general-purpose mechanism that does not tap internally represented domain-specific knowledge?

The first question assumes an explication of innateness in terms of *developmentalcanalization* that roughly corresponds to the degree of developmental rigidity of a trait in the face of variation across a range of environments (Ariew 1999; see also Mallon and Weinberg 2006). The second question assumes an explication of innateness in terms of *psychological primitiveness *(Cowie 1999; Samuels 2002), and asks whether the light-from-above prior is a domain-specific representation, whose acquisition cognitive science cannot explain.

Let’s consider the first question. Is the light-from-above prior developmentally rigid? Hershberger (1970) offered preliminary evidence that it is rigid. He showed that chickens reared in cages illuminated from below still behaved as though light was coming from above. From this result, Hershberger concluded that the light-from-above prior is developmentally rigid, and therefore is probably innate.

However, more recent psychophysical studies with human adults and children do not bear out this conclusion. These studies show that human observers’ light-from-above prior is *not* rigid to subtle variation in environmental and developmental circumstances. If ‘innate’ is understood as developmental robustness or rigidity, then these studies show that the light-from-above prior is *not* an innate psychological trait.

Adams et al. (2004) found that the light-from-above prior can be modified by repeated haptic feedback about the shape of an object. In their experiment, human adults made convex–concave judgements of bump-dent stimuli illuminated by a single light source. In making these judgements, they initially assumed the light source be roughly overhead, which enabled them to extract information about the shape of the stimuli from their shading. During a training phase, the same experimental participants made convex–concave judgements, while they were exposed to stimuli that appeared to be lit from the side. After each judgement, they received haptic feedback regarding shape, which reinforced the visual appearance that lighting came from the side. In a post training phase, when participants judged a set of visual stimuli identical to those in the initial condition, their “light prior” had shifted significantly from overhead towards the side, causing altered shape judgements. Furthermore, this acquired light-from-the-side prior was found to transfer to a different task, where observers judged which one of two sides of an oriented object was lighter in the absence of any evidence about the light-source.

In another experiment with human adults, Morgenstern et al. (2011) found that the light-from-above prior is also easily overridden by other sensory cues. In this study, experimental participants estimated the shape of an object from shading information. Morgenstern and colleagues inferred the ratio between the weight participants gave to their light-from-above prior and the weight they gave to lighting cues of different strength. It turned out that the prior accounted for very weak lighting information, as its impact could be quashed by barely perceptible lighting cues like low-contrast shadows.

Other studies in developmental psychology support the idea that Bayesian priors need not correspond to psychological traits that develop rigidly despite variability in environmental statistics. For example, Thomas et al. (2010) had 4- to 12-year-old children make convex/concave judgements for a shaded “polo mint” stimulus. They found an interaction between a light-from-above prior and a convexity prior that changed over the course of development: a convexity prior would have more weight early in childhood, while a light-from-above prior would have more weight only later on during puberty. Coherent with this conclusion is Stone’s (2011) result that the light-from-above prior is malleable throughout childhood. These results would be explained by the fact that “light does not come from a consistent direction relative to one’s own body (which is the frame of reference used in judging shape from shading until around 7 years of age) until children are able to walk” (Thomas et al. 2010, p. 6). So, the light-from-above prior is neither rigid nor is it invariant to variation in the statistical structure of the developmental environment. If we assume an analysis of innateness as developmental canalization, it is therefore unjustified to believe that the light-from-above prior must be an innate trait.

Let’s now consider the second question. Is the light-from-above prior a psychologically primitive domain-specific representation? There are three sets of considerations supporting a negative answer. First, domain-specific representations apply to a restricted class of entities and phenomena (Carey and Spelke 1994). However, Adams (2007) showed that the light-from-above prior applies to a relatively wide range of different entities and phenomena, as it would be engaged in visual search, shape perception, and reflectance judgement. This finding coheres with the idea that the light-from-above prior is *not* a psychologically primitive domain-specific representation, but is acquired through some general-purpose learning mechanism that is sensitive to the general predominance of overhead lighting.

Second, most Bayesian work in the psychophysics of perception assumes that priors such as the light-from-above prior have a particular univariate parametric form (e.g., a Gaussian distribution of one random variable associated with a specific environmental property), which might be taken to suggest that Bayesian priors must be bodies of domain-specific knowledge. However, in estimating shape from shading, at least two parameters are involved: one over lighting direction, another over shapes. Generally, these two parameters are assumed to be independent, which can make the learning tractable, ensuring that the estimated univariate prior distribution over lighting direction is the same as a joint distribution over lighting direction and shape.^5^ Independence properties ensure that the priors employed in much Bayesian cognitive science, particularly in psychophysics, are specific to a single parameter (or environmental property). But the independence assumption between parameters is generally unjustified, because several parameters are correlated with one another in the environment. Hence, many of the priors employed in Bayesian cognitive science should be understood as high-dimensional priors that may not be specific to any specific property in the environment. A high-dimensional prior spans many different parameters at the same time; and so, the class of situations is large, in which this very same prior can be recruited for acquiring novel psychological traits.

Third and finally, Bayesian priors like the light-from-above prior may themselves be acquired courtesy of some general-purpose mechanism that does not tap internally represented domain-specific knowledge. This means that Bayesian priors need *not* be psychologically primitive bodies of domain-specific knowledge. Clark (2013a, 2016 Sect. 6.3) makes this point by drawing on work by Kemp et al. (2007) and Tenenbaum et al. (2011). He writes: “multilayer Bayesian systems have proven capable of acquiring abstract, domain-specific principles without building in the kinds of knowledge [...] that subsequently account for the ease and efficacy of learning in different domains” (2013a, p. 488).

In hierarchical Bayesian systems, the hypothesis space has a hierarchical structure, and the only psychologically primitive, built-in knowledge is at the highest level of the hierarchy. This built-in knowledge is generic, as it corresponds to a *hyper-prior* on a *hyper-parameter*, which is a parameter of a prior distribution at the level below. While hyper-priors impose constraints on the kinds and range of representations the system can acquire, these constraints are increasingly weak as the number of levels in the system increases. “By adding further levels of abstraction to an HBM [hierarchical Bayesian model] while keeping pre-specified parameters to a minimum, at the highest levels of the model, we can come increasingly close to the classical empiricist proposal for the bottom-up, data-driven origins of abstract knowledge” (Perfors et al. 2011, p. 308; Perfors 2012). This means that hierarchical Bayesian systems that initially encode hyper-priors “concerning very abstract (at times almost Kantian) features of the world” (Clark 2013a, p. 487) can acquire domain-specific bodies of knowledge like the light-from-above prior by extracting structure from the environmental input (see Lee and Mumford 2003 for relevant neurophysiological evidence; for early neural networks that extract shape from shading see Lehky and Sejnowski 1988, 1990).

In summary, the initially given hyper-priors in a hierarchically organized Bayesian system are typically *not *domain-specific. If these hyper-priors are not domain-specific, and can explain how domain-specific representations like the light-from-above prior are acquired, then these representations are not psychologically primitive. If we assume an analysis of innateness as psychological primitiveness, it is therefore unjustified to believe that the light-from-above prior must be an innate trait.

Before moving on, there is something important to flag. Clark (2013a, b, 2016) and Tenenbaum et al. (2011) have Hierarchical Bayesian modelling in mind, when they claim that Bayesianism has fundamental consequences for the nativism versus empiricism debate. In Clark’s (2013a) words:

“Hierarchical Bayesian modelling shows that acquisition of psychological trait can proceed just as if it had been constrained by apt bodies of innate knowledge [...] it demonstrates that the potent, accelerated, domain-specific learning profiles often associated with such knowledge may also be displayed by systems that begin from much more minimal bases [...] The HBM accounts on offer share the singular virtue of accommodating many empiricist intuitions (for example, those concerning flexibility in the face of new environmental inputs) while leaving room for as much innate knowledge as well-controlled experimental studies may (or may not) eventually mandate” (p. 495).

The basic ideas are twofold: that Hierarchical Bayesian models equip the empiricist with previously unappreciated resources, and that Hierarchical Bayesian models have the singular virtue to provide both nativists and empiricists with a way of precisely assessing the relative contributions of both “innate, domain-specific knowledge” and “domain-general learning mechanisms” (Griffiths et al. 2008, p. 62).

I shall return to these ideas in Sect. 7. For now, suffices it to anticipate that the same enthusiasm was shown over 25 years ago, during the resurgence of Connectionism. Because connectionist models showed that data-driven induction and minimal initial biases could suffice to acquire novel, domain-specific bodies of knowledge (Clark 1993a; Elman et al. 1996), Connectionism was said to fundamentally reconfigure the debate between nativists and empiricists, which was shaped by Fodor’s (1975, 1981) and Chomsky’s (1980) Classical computationalism back then.

## Bayesian mechanisms between Nativism and Empiricism

Another common belief is that Bayesianism in cognitive science is committed to positing general-purpose learning mechanisms. In an influential review of Bayesian approaches in developmental psychology, Xu (2007, p. 214) asks: “Is the Bayesian inference mechanism domain-general, and if yes, in what sense?” She answers: “I have suggested... that this is not a mechanism specific to word learning, or language, or causal reasoning. However—she continues—I am not claiming that the same *token* of the Bayesian inference mechanism is used again and again in various domains. Rather Bayesian inference is a *type* of learning mechanism that can be instantiated many times over in the human brain/mind” (cf., Perfors et al. 2011, p. 316; Xu and Griffiths 2011).

When Xu (2007) describes Bayesian inference as “a type of learning mechanism,” she has in mind the Bayesian rule of conditionalization for computing posterior distributions. But this is not the mechanism that is actually involved in the accounts of word learning and causal reasoning she reviews. The types of mechanisms that are used to account for word learning and causal reasoning, and that might be tokened in the human “brain/mind,” cannot amount to simple conditionalization, because simple Bayesian conditionalization makes these learning tasks intractable. For tasks that involve high dimensionality, or complicated and unusual statistical structures, different types of mechanisms are required for updating “beliefs” and acquiring new psychological traits. So, Bayesian conditionalization and more specific types of Bayesian mechanisms should be kept distinct when we ask questions about the nature of Bayesian learning mechanisms.

Almost all accounts of the acquisition of high-level psychological traits, and of several low-level perceptual traits too, do not involve simple Bayesian conditionalization. They involve approximations like Monte Carlo and variational learning mechanisms, which can be tokened in a number of different ways depending on the problem in hand. We should examine the nature of these mechanisms, if we want to understand the possible implications of Bayesianism for the controversy between nativism and empiricism.

Some of the approximately Bayesian learning mechanisms that can tractably underlie the acquisition of psychological traits like the abilities to learn words or to acquire causal knowledge are more domain-specific than others. These mechanisms all employ precise probabilities to represent uncertainty, update probabilities in accord with the axioms of probability, and provide an approximation of the target posterior distribution. This is the only sense in which they are all Bayesian. There is no simply a Bayesian mechanism; but different species Bayesian mechanisms with a number of specific properties.

Bayesian decision theory offers a unifying mathematical language (Colombo and Hartmann 2017) along with a package of different methods for learning and inference. Depending on the details of the problem associated with the acquisition of a certain psychological trait—e.g., the size of the hypothesis space, the shape of the joint distribution over data and parameters, the time available to find a solution, and computational constraints on memory and search—Bayesianism can posit, equally plausibly, acquisition mechanisms that are domain-specific, or that are general-purpose. So, Bayesianism is not committed to positing a cognitive architecture that includes few general-purpose learning mechanisms that are ultimately responsible for the acquisition of psychological traits. I now sharpen and establish this conclusion by examining two species of Monte Carlo algorithms as mechanisms for category learning.^6^

## Bayesian mechanisms for acquiring categories

From a probabilistic perspective, the computational problem of category learning is to identify a probability distribution associated with each one of the available category labels. Category learning would consist of a problem of probability density^7^ estimation (Ashby and Alfonso-Reese 1995). Given the features (denoted by *d*) of an item, one should infer the category label *c* for that item from the set of available category labels *C*. For instance, given the features “has feathers,” “has wings,” “has a beak” for an item, one should infer the category label “Bird” from the set of available categories. Using Bayesian conditionalization, the posterior over category labels is:

2 $$\begin{aligned} P\left( {c{|}d} \right) = \frac{{P\left( {d{|}c} \right) P\left( c \right) }}{{\mathop \sum \nolimits _{{c' \in C}} P\left( {d{|}c^{\prime }} \right) P\left( {c'} \right) }} = ~\frac{{P\left( {d,~c} \right) }}{{\mathop \sum \nolimits _{{c' \in C}} P\left( {d,~c^{\prime }} \right) }} \end{aligned}$$

For categorization problems where the likelihoods can have any interesting structure, or where the space *C* of category labels is too large and complex, the posterior $P (c {\vert } d)$ cannot be computed tractably. For these problems, only approximations for the target posterior can be tractably computed. One class of mechanisms for computing such approximations consist of algorithms based on the Monte Carlo principle, which says that anything we want to know about a random variable $\theta$ can be learned by sampling many times from the distribution $P(\theta )$. This principle grounds a family of algorithms, which become increasingly accurate in approximating a target posterior distribution as the number of samples they use increase.

Two Monte Carlo algorithms that have been used to solve category learning problems are Gibbs sampling and particle filtering. The basic idea behind the Gibbs sampling algorithm is that joint probability densities can be characterised as component conditional densities. For all variables of a target joint distribution, the Gibbs sampler algorithm begins by selecting one variable—the order in which the Gibbs sampler selects variables does not affect its computations—then samples one value of that variable, and finally conditions the sampled value on the values of all other random variables and all data. Once all random variables are sampled, the Gibbs sampler has finished one iteration, which yields a distribution that approximates the target posterior. The accuracy of this approximation improves as a function of the number of iterations of the algorithm.

The basic idea behind the particle filter algorithm is that any joint probability density can be characterised as sets of samples (or particles) drawn from probability densities ‘related’ to the target joint probability density. For a target posterior distribution $P (\hbox {c}_{\mathrm{t}} {\vert } \hbox {d}_{1}, {\ldots }, \hbox {d}_{\mathrm{t}})$, the particle filter algorithm begins by generating a known ‘proposal distribution,’ which is related to the target one. For instance, the proposal distribution may be the prior probability $P (\hbox {c}_{\mathrm{t}} {\vert } \hbox {d}_{1}, {\ldots }, \hbox {d}_{\mathrm{t-1}})$. The algorithm first draws samples from the proposal distribution, then assigns each resulting sample a weight proportional to the probability that the sample comes from the target distribution. The same operation is repeated for all time steps *t*. Thus, the particle filter algorithm can get to approximate $P (\hbox {c}_{\mathrm{t}} {\vert } \hbox {d}_{1}, {\ldots }, \hbox {d}_{\mathrm{t}})$ by sampling and re-sampling from a sequence of related distributions $P (\hbox {c}_{\mathrm{t-1}} {\vert } \hbox {d}_{1}, {\ldots }, \hbox {d}_{\mathrm{t-1}})$, appropriately weighing each sample.

Both the Gibbs sampler and the particle filter algorithms are flexible, since they can be applied across different problem domains and can approximate complex, non-linear, non-Gaussian joint distributions. However, they differ in important ways. For example, the Gibbs sampler assumes that all data are available at the time of learning and inference: if new data arrive over the course of processing of the Gibbs sampler, then the Gibbs sampler must start its processing anew, which makes it unsuitable for online, sequential learning. Instead, the particle filter algorithm assumes that data are collected progressively over time: posterior distributions are approximated by propagating samples, whose weights are updated as a function of incoming observations, which makes particle filtering adapted to sequential environments. So, the proper domain of application of the Gibbs sampler corresponds to tasks where all data bearing on a certain hypothesis arrive simultaneously, while the particle filter algorithm is tailored to learning in dynamic tasks, where data arrive sequentially.

The Gibbs sampler, the particle filter, and other Bayesian algorithms were examined by Sanborn et al. (2010) as possible mechanisms for learning new categories. Firstly, Sanborn and colleagues characterised category learning in general, as a density estimation problem, where learners observe the features of a new item $\hbox {d}_{\mathrm{N}}$, and determine whether the label $\hbox {c}_{\mathrm{N} }=j$ applies to that item on the basis of all previous items, $\mathbf{d}_{\mathrm{N-1}}= (\hbox {d}_{1}$, $\hbox {d}_{2}, {\ldots }, \hbox {d}_{\mathrm{N-1}})$ and their labels $\mathbf{c}_{\mathrm{N-1} }= (\hbox {c}_{1}$, $\hbox {c}_{2}, {\ldots }, \hbox {c}_{\mathrm{N-1}})$.

Three general types of mechanisms for category learning were then distinguished. The first type corresponds to *parametric* learning algorithms, where the joint distribution *P* (***c***, ***d***) has some fixed parametric form. The second type corresponds to *non-parametric* learning algorithms, where the form of *P* (***c***, ***d***) is allowed to change as the amount of data is increased. Importantly, parametric algorithms make stronger assumptions about the shape of *P* (***c***, ***d***). Non-parametric algorithms do not assume any specific family of distributions for the category structure *P* (***c***, ***d***).

As Ashby and Alfonso-Reese (1995) point out, parametric learning mechanisms are naturally associated with prototype-based category learning, while non-parametric mechanisms are associated with exemplar-based category learning. So, insofar as we learn categories on the basis of both exemplars and prototypes, our cognitive architecture may well include both parametric and non-parametric Bayesian learning mechanisms.

A third, intermediate, type of learning mechanism discussed by Sanborn and colleagues assumes that categories are broken down into several clusters $\mathbf{z} = (\hbox {z}_{1}$, $\hbox {z}_{2}, {\ldots }, \hbox {z}_{\mathrm{N}})$; each cluster z is assigned a parametric distribution and the category distribution becomes a *mixture model* associated with the joint distribution $P({{\varvec{c}}}, {{\varvec{d}}},{{\varvec{z}}})$, where each cluster is represented by a parametric distribution, and the full joint distribution is represented by a mixture of those distributions.

Sanborn and colleagues focused on a mixture model for the task of category learning: the *Dirichlet process mixture model* (DPMM), where Dirichlet distributions are used as prior distributions in the model, which allows capturing a broad range of densities. Sanborn et al. (2010) compared the degree of fit of different learning mechanisms, including a Gibbs sampler and a particle filter, with human performance in several category learning tasks understood as DPMM. While they found that a particle filter algorithm had an especially good fit to humans’ learning of categories, their study bears out three conclusions: That different Bayesian learning mechanisms have different degrees of domain-specificity, where the notion of a domain should not be understood in terms of a subject matter; that the degree of domain-specificity of different Bayesian mechanisms depends on the types of assumptions they make about the statistical or temporal properties of a target problem, and that the Bayesian approach can posit, equally plausibly, learning mechanisms that are domain-specific, or else more general-purpose, for the acquisition of the same types of psychological traits.

Different Bayesian learning algorithms make different assumptions about the type of process that generates the data in a task of interest. Such assumptions are captured by different generative models, which specify a joint probability distribution over data and hypotheses—for instance, a joint probability *P* (***c***, ***d***) over features of an item to categorise and sequences of category labels. The degree of domain-specificity of a Bayesian learning algorithm depends on the extent to which the algorithm is constrained to process data assumed to be produced by a generative process with a fixed, specific statistical form.

Parametric learning algorithms make stronger assumptions than non-parametric algorithms about the family of the probability distribution from which data are generated, though they are generally less memory and time consuming than non-parametric learning algorithms. Given these stronger assumptions, the algorithm is bound to yield a posterior with a specific form, regardless of the amount of data observed. So, parametric Bayesian learning algorithms are more domain-specific than non-parametric ones: they are constrained to process inputs of a specific sort, inputs assumed to be produced by a generative process associated with a fixed statistical form.

Furthermore, distinct Bayesian algorithms for estimating posterior distributions, like the Gibbs sampling and the particle filter algorithms, are tailored to input data with different statistical and temporal properties. The Gibbs sampling algorithm can run to approximate a target posterior distribution only if all relevant data are available at a time. Thus, while a Gibbs sampler might underlie our ability to learning categories when all relevant exemplars are stored in memory or available at a time, it is fit to capture category effects on reconstruction from memory (Shi et al. 2010). The particle filter algorithm is specifically fit to process sequential data, and to update probability distributions over time. Thus, a particle filter algorithm may underlie our ability to learning categories when exemplars are revealed sequentially; and because it is tailored to sequential data, a particle filter algorithm can easily capture order effects when new pieces information are encountered over time (Sanborn et al. 2010).

## What’s next: back to the future?

There is a déjà-vu, when we read the claims made by Clark (2013a, b, 2016), by Tenenbaum et al. (2011) and by other enthusiasts of the Bayesian approach in cognitive science, about how this approach fundamentally reconfigures the dialectic between nativists and empiricists. The same types of claims were made over 25 years ago, when Connectionism re-emerged as a serious alternative to Classical computationalism. While Connectionism and Classicism are not the only alternatives to Bayesianism as approaches to explaining the acquisition of psychological traits, the Classicism vs Connectionism debate is a useful baseline for assessing the novelty and significance of the contribution that Bayesian cognitive science can make to the nativism/empiricism debate.

Now, just like Bayesianism, Connectionism was said to have fundamental implications for our understanding of the debate between nativists and empiricists, which back then was shaped by Fodor’s (1975, 1981, 1983) and Chomsky’s (1980, 1988) Classical ideas about the architecture of cognition (cf., Ramsey and Stich 1991; Karmiloff-Smith 1992; Clark 1993a; Quartz 1993; Elman et al. 1996). By the late 1970s, the Connectionist approach began to show how psychological traits can be acquired gradually and gracefully, courtesy of associative learning algorithms applied to a rich body of data, without the need to posit explicit rules and a system of atomic, domain-specific, representational states with combinatorial syntactic and semantic structure (aka an innate *language of thought*). Connectionism demonstrated how novel psychological traits could emerge from error-driven changes in patterns of activation within artificial neural networks, whose architectures need not include a pre-wired language of thought.

Connectionist models demonstrated that language, face-recognition abilities, categorization, and many other psychological traits that were previously thought to be un-learnable, could be acquired courtesy of the interplay between the statistics of the environment, the knowledge embodied in the initial state of a neural network, and error-driven learning algorithms applicable to several different psychological domains (Elman et al. 1996; but see Fodor and Pylyshyn 1988 for an influential criticism). While the connectionist approach is not intrinsically anti-nativist, it offered one way to think about how the developmental trajectory of psychological traits depends “on the nature of the statistical structure present in everyday experience, and how this structure is exploited by learning” (Rogers and McClelland 2014, p. 1041).

Connectionism opened up a space of possible nativisms associated with different pre-setting of connection weights and pre-structuring of different architectures (Ramsey and Stich 1991; Clark 1993a; Quartz 1993). In particular, Connectionism uncovered a *minimal form of rationalism*, which Clark (1993b) characterised as follows:

“Instead of building in large amounts of innate knowledge and structure, build in whatever minimal set of biases and structure will ensure the emergence, under realistic environmental conditions, of the basic knowledge necessary for early success and subsequent learning.” (p. 598)

Minimal rationalism straddled accepted categories in the debate. It posited weak, initial biases and algorithmic transformation factors, which could filter incoming data with different statistical and temporal properties for processing in specific circuits in a network. This complex interaction between weak architectural biases, transformation factors, and external statistical structure could tug learning towards novel psychological traits, including novel, domain-specific bodies of knowledge.

Cowie (1999) makes a similar point about Connectionism, after she distinguishes *Chomskyan Nativism* from *Weak Nativism* and *Enlightened Empiricism *as logically possible positions about language learning. *Chomskyan Nativism* is committed to three ideas: (DS) that learning a language requires bodies of knowledge specific to the linguistic domain; (I) that the bodies of knowledge constraining learners’ thoughts during language learning are innate, in the sense that they are psychologically primitive; and (UG) that the bodies of knowledge specified in (DS) as being required for language learning are the principles of the Universal Grammar (Cowie 1999, p. 176). *Weak Nativism* accepts (DS) and (I), but rejects (UG); *Enlightened Empiricism* accepts (DS), but rejects (I) and (UG). At various points in her treatment, Cowie suggests that at least some early connectionist learning algorithms underwrite *Enlightened Empiricism*, since they would display a mechanism that is both general-purpose and able to learn a language by making use of domain-specific knowledge acquired along the way (Cowie 1999, pp. 234ff; 281ff).

Although Bayesianism and Connectionism differ in aspirations as well as in the kinds of acquisition mechanisms and representational structures they can posit (Griffiths et al. 2010; McClelland et al. 2010), both approaches show how data-driven inductive algorithms and weak initial biases can lead to the acquisition of a wide variety of psychological traits. Like Connectionism, Bayesianism holds that cognitive development is driven by patterns of prediction errors about the statistical structure of the environment, which constrains the space of possible trajectories of cognitive development (Téglás et al. 2011). Like Connectionism, Bayesianism can posit a wide variety of initial biases and algorithms that can tug learning in appropriate directions avoiding being held hostage of the statistics of the environmental input (Austerweil et al. 2015).

Most importantly, like Connectionism, Bayesianism *coheres with*, but does not entail, a form of *minimal rationalism* (or *enlightenedempiricism*), exactly of the type Clark (1993a) and Cowie (1999) singled out as serious alternatives to Fodorian and Chomskyian nativism. For example, Goodman et al. (2011) use the label ‘minimal nativism’ to describe how causal understanding can be acquired courtesy of a hierarchical Bayesian learning mechanism paired with innate bodies of abstract, generic knowledge, and with a collection of domain-specific mechanisms for analysing perceptual input.

Despite these analogies, Clark (2013a) identifies two problems with the Connectionist approach that would highlight one way of understanding how the relevance of Bayesianism for the nativism debate is novel and distinct. First, early connectionist models required fully supervised learning algorithms. Second, early connectionist models handled multilayer forms of learning with difficulty.

According to Clark (2013a), the Bayesian approach—Hierarchical Bayesian Modelling (HBM) in particular—avoids both problems. Unlike Connectionism, the Bayesian approach would show how unsupervised and self-supervised forms of hierarchical learning can be responsible for the quick, robust, and smooth acquisition of novel psychological traits. Stacking prior probabilities over prior probabilities in a hierarchically organized Bayesian model would be the key to this form of learning. Tenenbaum et al. (2011) explain that “each degree of freedom at a higher level of a HBM influences and pools evidence from many variables at levels below” (p. 1284). Hyper-priors in a HBM—that is, prior distributions on the parameters of a prior distribution at the lower level in the hierarchy—allow for potentially more complex hypotheses spaces be searched; HBM would also disclose a fast, robust, data-driven route to the acquisition of novel high-level psychological traits and abstract, domain-specific principles.

However, the concern is somewhat misplaced that early connectionist models required a teaching signal and could handle multi-layer (or hierarchical) learning only with difficulty. On the one hand, it was clear already in the 1980s that both auto-encoders (aka auto-associators) and recurrent networks that relied on back propagation need not require labelled training data. These networks could successfully carry out their processing in a self-supervised fashion by learning a compact, invertible code that allowed them to reconstruct their own input on their output (i.e., the target output of an auto-encoder is the input itself) (Hinton 1989, p. 208).

On the other hand, multi-layer forms of learning in early connectionist models were not precluded, although they were indeed harder to obtain in comparison to recent advances in deep learning (Hinton 2014). For instance, Boltzmann machines (Hinton and Sejnowski 1986) and restricted Boltzmann machines (Smolensky 1986) were early neural networks that could learn hierarchies of progressively more abstract and complex domain-specific representations without the need of any labelled data. As Clark (1993b) himself noted, pattern associators can acquire highly theoretical knowledge. Specifically, “multilayer nonlinear networks may develop highly abstract feature spaces in which continued processing is oblivious to many features of the concrete input. Such feature spaces may be the homes of a variety of different orders of prototype-based representation” (p. 103).

While the bearing of Bayesianism on the controversy between nativists and empiricists largely recapitulates that of Connectionism in these respects, Bayesianism might be thought to contribute a more transparent account of cognitive change in comparison to Connectionism. This transparency has two aspects. First, the representational and algorithmic assumptions made by Bayesian models are explicit: the space of the hypotheses under considerations, the prior probability of each hypothesis, and the relation between hypotheses and data are transparent. This transparency makes it relatively easy to understand what shapes a model’s behaviour, and why it fails or succeeds in accounting for the acquisition of a psychological trait (Griffiths et al. 2010, p. 358). Connectionist networks are generally more opaque, since it can be difficult to understand what exactly drives cognitive change, and which conditions are necessary for a certain psychological trait to emerge (Rogers and McClelland 2014, pp. 1056–1057).

So, in comparison to connectionist networks, Bayesian models make it easier for cognitive scientists to formulate and evaluate explicit hypotheses concerning the kind of innate structure required for acquiring new psychological traits. For they make it more transparent and precise what problem a learner is supposed to solve, what kinds of primitive representational resources are available to the learner (Are these primitive representations domain-specific or not? What are the hypotheses actively represented and manipulated by the learner?), and what kinds of learning mechanisms the learner can use in order to acquire new psychological traits in environments with different statistical structures (Are the learning mechanisms general-purpose? Can they flexibly learn structures with different shapes? What features of the data can influence their processes?). At the very least, then, Bayesianism helps steer clear of pointless controversies that merely stem from the opacity of the causes of a model’s behaviour.

The transparency of Bayesianism has a second aspect too. Bayesian systems are more semantically transparent than Connectionist ones, where a system is “*semantically transparent* just in case it is possible to describe a neat mapping between a symbolic (conceptual level) semantic description of the system’s behavior and some *projectible *semantic interpretation of the internally represented *objects of *its formal computational activity” (Clark 1989, p. 18). According to this idea, the representational posits of Bayesianism, but not of Connectionism, can be related in a systematic way to features of the world that can be picked out propositionally, with the expressive resources of public language.

The higher degree of semantic transparency of Bayesian models should not surprise us, since Bayesianism is perhaps the best developed account of *rational* degrees of *belief*. Although hypotheses in Bayesian models can take any form, in practice they often correspond to causal graphs, sometimes they correspond to distributed patterns of activation in a neural network, and, most interestingly here, they can also consist of structured symbols in a probabilistic language of thought (Ullman et al. 2012; Goodman et al. 2015). When a Bayesian system embodies structured, symbolic representations, it becomes transparent how to evaluate the *rationality* of its cognitive change and development. For it allows us to pick out the probabilistic and logical relationships between its representations, and to evaluate their probabilistic and deductive coherence, both synchronically and diachronically.

*Rational constructivism* is in fact another label that has been used to characterise Bayesianism (Xu 2007; Xu and Griffiths 2011; Xu and Kushnir 2013). Rational constructivism is committed to three ideas: (a) that the learning mechanisms that best explain cognitive change and developmental are domain-general Bayesian mechanisms, which may give rise to domain-specific knowledge; (b) that innate (i.e. psychologically primitive) representations need not include just non-conceptual bodies of knowledge, but may include representations of logical operators such as *and/or/all/some*, representations of variables, and logically richer representations too; and (c) that “the construction of new concepts and new learning biases is driven by rational inferential learning processes” (Xu and Griffiths 2011, p. 299). These rational inferential learning processes would display learning as a kind of theory construction realized as Bayesian hypothesis testing, and would contrast with the associative learning processes of connectionist networks, which classicists like Fodor conceive of as non-rational, brute-causal processes.

With this higher degree of semantic transparency, Bayesianism allows for a sharp distinction between implementation, algorithm and representation, and computational function; a distinction that resonates with Classical treatments of the nativism debate, but was eroded within Connectionism. Unlike connectionist models, where the distinction between implementation and function is effectively eroded, both Classicist and Bayesian models do not obviously allow for understanding how structural alterations in the architecture of a system may have functional consequences for the representational power of the system (Quartz 1993, p. 234). If this is correct, then Bayesianism not only recapitulates some of the implications that Connectionism had for the nativism versus empiricism debate over 25 years ago, but, ironically, Bayesianism can also salvage some Classical ideas concerning the relations between distinct levels of cognitive analysis (Marr 1982), and about the rational, productive, and systematic nature of thinking and learning (Fodor and Pylyshyn 1988).

## Conclusion

Bayesianism offers a fertile source of modelling tools rather than a well-understood and empirically supported theory of the innate architecture of the human mind. A substantial account of the acquisition of a given psychological trait should be able to answer a number of questions. For example, are we talking of an evolved trait, or of a trait culturally acquired during development, or of a trait acquired through a developmental process triggered by a narrow range of variation in environmental conditions? And what kinds of learning mechanisms and representations are necessary for the acquisition of the trait? While Bayesianism alone cannot answer these questions, it can frame them in a precise and transparent way, combining aspects of both Connectionism and Classicism. Like Connectionism, Bayesianism shows that an innate language of thought is not required to account for the acquisition of high-level psychological traits. Like Classicism, Bayesianism offers a transparent way of evaluating the character of the innate structure in the human cognitive architecture. Combining these aspects, what Bayesianism brings to the table is not a vindication of either nativism or empiricism, but one flexible and precise way to transparently capture and evaluate the character of the innate structure in the human cognitive architecture.

## Footnotes

1 Not all Bayesian models are meant to make substantial claims about the mechanisms and representations underlying cognition and behaviour. Some Bayesian models are meant to offer only an encompassing mathematical template that can be applied to a wide range of phenomena in order to provide computational-level analyses (Anderson 1990; Marr 1982) and/or in order to unify these phenomena without making commitments to underlying mechanisms and representations (Colombo and Hartmann 2017; Danks 2014, Ch. 8). Following Clark (2013a, b) and Samet and Zaitchik (2014, note 28), here I set aside questions about the psychological reality of Bayesian models (Colombo and Seriès 2012). Rather, I assume that Bayesianism offers not only a mathematical template or computational-level analyses. Bayesianism can also make substantial empirical claims about the nature of learning mechanisms and representations (cf., Hohwy 2013; Clark 2013b, 2016).

2 Bayes’ *theorem *is a provable mathematical statement that expresses the relationship between conditional probabilities and their inverses. Bayes’ theorem expressed in odds form is known as Bayes’ *rule*. The rule of *conditionalization* is a prescriptive norm that dictates how to reallocate probabilities in light of new evidence or data.

3 It is worth pointing out that Bayesianism is by no means the only theory of learning and decision-making under uncertainty (see Colombo et al. 2016 for a critical treatment of Bayesianism in cognitive science and its possible alternatives).

4 Some treatments in Bayesian cognitive science (e.g., Seydell et al. 2011) assume an explication of *innateness* in terms of adaptation, according to which innate traits are those whose acquisition can only be explained by natural selection. Assuming this explication, these treatments associate the question of whether Bayesian priors are innate with the question of whether Bayesian priors match the statistics of natural environments. The idea is that if a prior like the light-from-above prior matches the relative frequency of light sources coming from overhead, then the prior can be explained by natural selection, and so is probably innate. However, the fact that the light-from-above prior, and other well-studied Bayesian priors like the slow-speed prior (Stocker and Simoncelli 2006), might reflect natural frequencies in the environment (Simoncelli and Olshausen 2001) does not warrant the inference that they are explained by natural selection, nor does it warrant the inference that Bayesian priors *should* be tuned to natural frequencies (Feldman 2013; for a compact discussion of this point see Sotiropoulos and Seriès 2015, Sect. 3.4).

5 More precisely, the assumption of parameter independence implies that the marginal distribution is the same as the conditional distribution.

6 This point could only be strengthened if alternative types of mechanisms are also examined that involve, for example, the Laplace approximation or variational algorithms.

7 For ease of presentation, I use the terms ‘probability density’ and ‘probability distribution’ interchangeably, although the two concepts are distinct. Roughly, a probability density function is a function that describes the relative likelihood for a continuous random variable to take on a given value.
